# Critical Role of FLRT1 Phosphorylation in the Interdependent Regulation of FLRT1 Function and FGF Receptor Signalling

**DOI:** 10.1371/journal.pone.0010264

**Published:** 2010-04-22

**Authors:** Lee M. Wheldon, Bryan P. Haines, Rajit Rajappa, Ivor Mason, Peter W. Rigby, John K. Heath

**Affiliations:** 1 Molecular Bacteriology and Immunology Group (MBIG), Centre for Biomolecular Sciences, University of Nottingham, Nottingham, United Kingdom; 2 Section of Gene Function and Regulation, The Institute of Cancer Research, Chester Beatty Laboratories, London, United Kingdom; 3 MRC Centre for Developmental Neurobiology, King's College London, London, United Kingdom; 4 Cancer Research UK Growth Factor Group, School of Biosciences, University of Birmingham, Birmingham, United Kingdom; University of Southampton, United Kingdom

## Abstract

**Background:**

Fibronectin leucine rich transmembrane (FLRT) proteins have dual properties as regulators of cell adhesion and potentiators of fibroblast growth factor (FGF) mediated signalling. The mechanism by which the latter is achieved is still unknown and is the subject of this investigation.

**Principal Findings:**

Here we show that FLRT1 is a target for tyrosine phosphorylation mediated by FGFR1 and implicate a non-receptor Src family kinase (SFK). We identify the target tyrosine residues in the cytoplasmic domain of FLRT1 and show that these are not direct substrates for Src kinase suggesting that the SFK may exert effects via potentiation of FGFR1 kinase activity. We show that whilst FLRT1 expression results in a ligand-dependent elevation of MAP kinase activity, a mutant version of FLRT1, defective as an FGFR1 kinase substrate (Y3F-FLRT1), has the property of eliciting ligand-independent chronic activation of the MAP kinase pathway which is suppressed by pharmacological inhibition of either FGFR1 or Src kinase. Functional investigation of FGFR1 and FLRT1 signalling in SH-SY5Y neuroblastoma cells reveals that FLRT1 alone acts to induce a multi-polar phenotype whereas the combination of FLRT1 and FGFR activation, or expression of Y3F-FLRT1, acts to induce neurite outgrowth via MAPK activation. Similar results were obtained in a dendrite outgrowth assay in primary hippocampal neurons. We also show that FGFR1, FLRT1 and activated Src are co-localized and this complex is trafficked toward the soma of the cell. The presence of Y3F-FLRT1 rather than FLRT1 resulted in prolonged localization of this complex within the neuritic arbour.

**Conclusions:**

This study shows that the phosphorylation state of FLRT1, which is itself FGFR1 dependent, may play a critical role in the potentiation of FGFR1 signalling and may also depend on a SFK-dependent phosphorylation mechanism acting via the FGFR. This is consistent with an ‘in vivo’ role for FLRT1 regulation of FGF signalling via SFKs. Furthermore, the phosphorylation-dependent futile cycle mechanism controlling FGFR1 signalling is concurrently crucial for regulation of FLRT1-mediated neurite outgrowth.

## Introduction

Knowledge of the architecture of receptor tyrosine kinase signalling pathways is rapidly expanding but much less is known about the mechanisms that shape the spatial and temporal dynamics of signal propagation. In particular, a number of agents have been identified which attenuate or accelerate signalling through downstream pathways [1] but their mechanisms of action are frequently poorly understood. Here we focus on the fibronectin leucine rich transmembrane proteins (FLRTs): a subclass of the larger diverse leucine rich repeat (LRR) superfamily [2] which act as multifunctional accelerators of fibroblast growth factor receptor (FGFR) signalling. We, and others, have demonstrated that: members of the FLRT family associate with members of the FGFR family, accentuate FGF-mediated signalling via the Ras/Raf/ERK pathway and play a role in cadherin-dependent homotypic cell adhesion functions [3], [4], [5]. A key issue in further understanding the function of FLRTs is to determine the inter-relationships between these three cardinal properties.

Three members of the FLRT family (FLRT 1–3) have been identified in higher vertebrates from functional screens and in silico searches [6]. They exhibit canonical fibronectin and leucine rich repeat motifs in the extracellular domain which mediate the homotypic cell adhesion functions; a single transmembrane domain and a short (∼100 amino acid) cytoplasmic domain devoid of overt signalling motifs. Each FLRT family member exhibits characteristic and restricted patterns of expression in the developing embryo [3], [4], [7]. FLRT1, the subject of this study, is expressed in adult brain and kidney [6] and, in embryonic development, is localized in the midbrain at the boundary with the hindbrain and in the dorsal diencephelon adjacent to the telencephalon, the eye, dorsal root and trigeminal ganglia and in cells adjacent to the urogenital ridge [4]. This pattern overlaps with regions of FGFR and FGF ligand expression suggestive of a specific requirement for interaction of the FGF and FLRT axis in these cell types. Indeed a potential role for FLRT action in neuronal function has been proposed from studies of FLRT3 expression in neural regeneration models [8], [9], [10].

In this work we set out to further understand the functional relationship between FGFR activation and FLRT function via an initial analysis of FGFR-mediated phosphorylation of FLRT1. We show that phosphorylation of FLRT1 in the cytoplasmic domain modulates the ability of FLRT to activate the MAPK pathway and induce neurite outgrowth. A non-phosphorylated form of FLRT1 acts as a chronic activator of FGFR1 signalling and both signalling propagation and induction of neurite outgrowth require the activity of a non-receptor Src family kinase.

## Results

### FLRT1 and FGFR1 are co-localized

**Figure 1:**
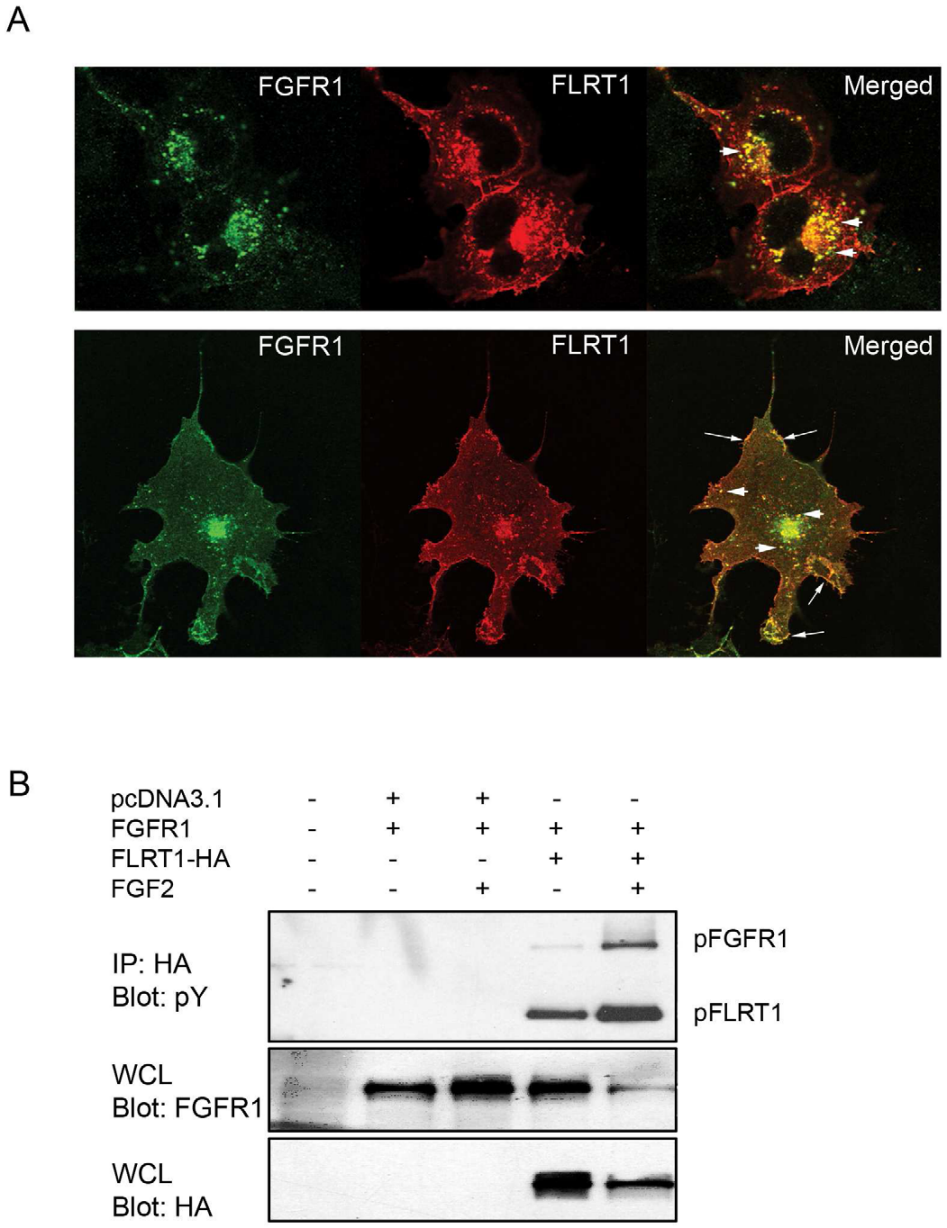
Co-localization of FLRT1 and FGFR1. A) Immunofluorescent staining of Cos-7 cells co-transfected with plasmids encoding FGFR1 and 3'HA-tagged FLRT1 Cells were stained with anti-FGFR1 (green) and anti-HA (FLRT1 -red) Merged images show areas of co-localisation in yellow Images (in section) were taken with a confocal microscope and are representative cells from 11 total fields of cells B) HEK 293T cells were co-transfected with FGFR1 and either control vector (pcDNA31) or FLRT1 (FLRT-HA) with or without stimulation with FGF2 (20ng/ml) in the presence of heparin (10mg/ml) for 30 min Anti-HA immunoprecipitation was performed on whole cell lysate which was subjected to western blot analysis with anti-phosphotyrosine (IP: HA, Blot: pY) to identify phosphorylated FLRT1 (pFLRT1) Phosphorylated FGFR1 (pFGFR1) was co-immunoprecipitated with FLRT1 The whole cell lysate (WCL) was probed for both FGFR1 (Blot: anti-FGFR1) and FLRT1 (Blot: anti-HA) expression Data is representative of at least 4 independent experiments.

We have previously documented an association between FGFR1 and FLRT1 [4] and we were interested to learn the cellular location(s) of this interaction. Cos-7 cells transiently co-transfected with FGFR1 and FLRT1 demonstrated clear co-localisation in punctate perinuclear intracellular vesicles (Figure 1A, thick white arrows, upper and lower panels) and at the cell surface membrane (Figure 1A, thin white arrows, lower panels). This data shows that FGFR1 and FLRT1 localise to similar multiple cellular compartments.

### FGFR1 phosphorylates FLRT1

This pattern of co-localisation raised the possibility that FLRT1 could be a potential substrate for ligand-mediated FGFR1 phosphorylation which could, in principle, regulate FLRT function. Co-transfection of FGFR1 and FLRT1 in 293T cells results in robust ligand-independent tyrosine phosphorylation of FLRT1 (Figure 1B), probably as a consequence of elevated receptor population and ligand-independent FGFR1 activation, showing that either FGFR1 itself or other downstream kinases can utilise FLRT1 as a substrate.

**Figure 2:**
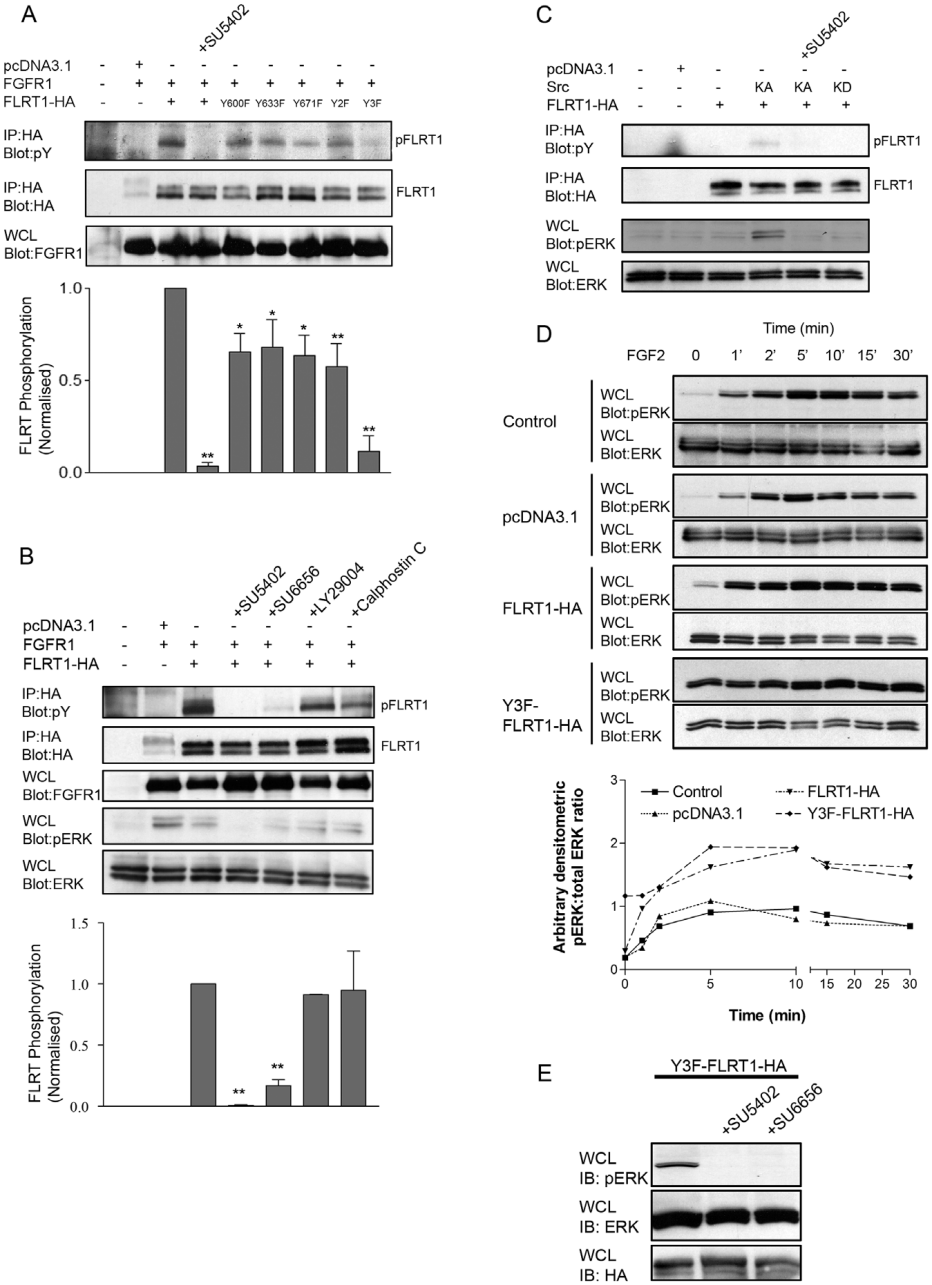
FLRT1 is not a SFK substrate but phosphorylation is FGFR-and SFK-dependent. A) HEK 293T cells were transfected with control (pcDNA31) or FGFR1 and a panel of either full-length FLRT1-HA or tyrosine substitution contructs as indicated (see Materials & Methods) One sample was pre-treated with FGFR kinase inhibitor (SU5402, 50mM, 1 hr) where indicated Cell lysates were immunoprecipitated with anti-HA and subsequently blotted with anti-phosphotyrosine (IP: HA, Blot: pY) or anti-HA (IP: HA, Blot: HA) to examine phosphorylated FLRT1HA levels (pFLRT1) or total immunoprecipitated FLRT1-HA levels (FLRT1), respectively Whole cell lysate (WCL) fractions were probed with anti-FGFR1 (Blot: FGFR) to control for protein expression B) HEK 293T cells were transfected with pcDNA31, FGFR1 and FLRT1-HA as indicated Cells were pre-incubated (1hr) with pharmacological inhibitors (SU5402, 50mM; SU6656, 20mM) Cell lysates were immunoprecipitated with anti-HA and subsequently blotted with anti-phosphotyrosine (IP: HA, Blot: pY) for pFLRT1 and anti-HA (IP: HA, Blot: HA) for FLRT1 WCL fractions were probed with anti-FGFR1 (Blot: FGFR), anti-phospho-ERK (Blot: pERK) or anti-ERK (Blot: ERK) Data in A) and B) are representative of ≥3 independent experiments Densitometric analysis (mean ± sem, n = 3) is the ratio of pFLRT1:FLRT1 and normalised to FLRT1 phosphorylation in the absence of inhibitor in both cases (**p<001, *p<005 non-parametric one way ANOVA) C) HEK 293T cells were transfected with pcDNA31, FLRT1-HA alone or co-transfected with either a constitutively active (KA) or kinase dead (KD) c-Src construct Cells were serum starved for 1hr and cell lysates immunoprecipitated with anti-HA and subsequently blotted with anti-phosphotyrosine (IP: HA, Blot: pY) for pFLRT1 or anti-HA (IP: HA, Blot: HA) for FLRT1 WCL fractions were probed with anti-phospho-ERK (Blot: pERK) or anti-ERK (Blot: ERK) Data are representative of 3 independent experiments D) HEK 293T cells were transfected with either pcDNA31 vector, FLRT1-HA or Y3F-FLRT1-HA constructs Cells were co-stimulated with FGF2 (20ng/ml) and heparin (10mg/ml) for the indicated times Cell lysates were blotted for anti-phospho-ERK (WCL IB: pERK), membranes were stripped and re-probed for anti-ERK (WCL IB: ERK) Densitometric analysis has been adjusted for ERK loading and expressed as an arbitrary pERK:ERK ratio Data are representative of at least 4 independent experiments E) 293T cells transfected with Y3F-FLRT1-HA were serum-starved in the absence or presence of pharmacological inhibitors of FGFR1 (SU5402, 50mM) and SFKs (SU6656, 20mM) and whole cell lysates probed with antiphospho-ERK (IB: pERK), anti-ERK (IB: ERK) and anti-HA (Blot: HA) for Y3FFLRT1-HA.

Bioinformatic analysis of theoretical tyrosine phosphorylation sites on the C-terminal region of FLRT1 (NetPhos 2.0 http://www.cbs.dtu.dk/services/NetPhos/) revealed 3 high probability residues, Y600, Y633 and Y671. A panel of single, double and the triple tyrosine substitution constructs was produced and examined for expression and localisation in transfected 293T cells. In all cases, protein was localized at both the plasma membrane and in intracellular vesicular-like structures (data not shown) suggesting that mutation of these cytoplasmic tyrosine residues to phenylalanine did not grossly perturb FLRT1 expression or intracellular trafficking. Mutation of these tyrosine residues in FLRT1 decreased FGFR1-mediated FLRT1 tyrosine phosphorylation compared to wild -type in all cases (Figure 2A). The single tyrosine deletion constructs Y600F-FLRT1, Y633F-FLRT1, Y671F-FLRT1 and the double mutant (Y600, 633F) Y2F-FLRT1 exhibit reduced, but not abolished, phosphorylation (∼34%, ∼32%, ∼36% and ∼42% inhibition, respectively) whereas the triple mutant (Y3F-FLRT1) exhibited almost complete abolition of tyrosine phosphorylation (Figure S1) equivalent to that observed by pharmacological inhibition [11] of FGFR1 kinase with SU5402 (∼88% and ∼96% inhibition, respectively). These results establish that Y600 , Y633 and Y671 are critical for FGFR1-mediated phosphorylation of FLRT1, each site is phosphorylated and FGFR1 activation is necessary and sufficient for FLRT1 phosphorylation. These findings also predict that Y3F-FLRT1 is defective in a process (or processes) which require phosphorylation of the 3 critical tyrosines.

We next sought to further characterise the kinase(s) responsible for FGFR1 mediated phosphorylation of FLRT1 using a pharmacological approach (Figure 2B). We were particularly interested in the role of SFKs in view of our previous findings [12], [13] that Src activation is a consequence of FGFR1 activation and Src activity is required for both receptor activation and mediation of downstream signalling dynamics. We observed, as before, almost complete inhibition of FLRT1 phosphorylation in the presence of SU5402 (Figure 2B). We also observed significant (83%) inhibition in the presence of the SFK inhibitor SU6656 [14] (Figure S1) comparable to that observed in the Y3FFLRT1 deletion construct (∼83% compared to ∼88%). In parallel ERK activation was significantly reduced in the presence of SU5402 or SU6656 which reflected the effect on FLRT1 phosphorylation. These data confirm that FLRT1 phosphorylation is FGFR-dependent and implicate an SU6656-sensitive SFK in the phosphorylation mechanism.

Since Src kinase acts both upstream and downstream of FGFR1 activation we next tested whether FLRT1 was a direct substrate for Src. Constitutively active (KA-Src (Y527F) and kinase-dead (KD-Src (K295M/Y527F)) Src were co-expressed with FLRT1 which was tested for tyrosine phosphorylation (Figure 2C). This revealed a very low degree of FLRT1 phosphorylation (even after 100× longer than normal exposure times) which was suppressed by the FGFR1 kinase inhibitor SU5402, as was the ability of KA-Src to activate ERK via phosphorylation. We conclude from these results that FLRT1 is a poor substrate for Src kinase and that Src regulates FLRT phosphorylation indirectly by virtue of the ability of KA-Src to activate FGFR signalling in the absence of ligand [12].

### Y3F-FLRT1 induces chronic ERK activation

Having established that FLRT1 is a target for FGFR1-mediated phosphorylation we sought to examine the role of FLRT1 phosphorylation in the FGF-mediated ERK response [3] from an endogenous FGFR population. 293T cells were transfected with either FLRT1 or Y3F-FLRT1 triple mutant form and tested for the dynamics of ERK activation in response to FGF stimulation (Figure 2D). In accord with previous results, FLRT1 expression enhanced the FGF response both at early (1min) and later time points (30 min). Much to our surprise, expression of Y3F-FLRT1 resulted in chronic stimulation of the ERK pathway both in the absence and presence of ligand. These results suggested that the phosphorylation-defective form of FLRT1 emulated the action of FGF in activating ERK signalling. Given that FLRT1 and FGFR1 coassociate we reasoned that the action of Y3F-FLRT1 might arise from activation of FGFR1. 293T cells were transfected with Y3F-FLRT1 and tested for ERK activation in the presence of pharmacological inhibitors of FGFR kinase (SU5402) and Src (SU6656). This revealed (Figure 2E) that the ability of Y3F-FLRT1 to elicit ERK activation is completely dependent on both FGFR and Src family kinase activity.

Thus the biochemical evidence reveals that FLRT1 is a target for Src-dependent FGFR-mediated phosphorylation and abolition of FLRT phosphorylation, by mutation of the substrate tyrosine residues, resulted in chronic ligand-independent yet FGFR1dependent ERK activation. This suggests a futile cycle relationship between FLRT1 and FGFR1 which is mediated by Src. The non-phosphorylated form of FLRT1 may activate FGFR1 via Src [12] resulting in phosphorylation of FLRT1.

### FLRT1 promotes neurite outgrowth in SH-SY5Y cells

Having established a functional interaction between FGFR1 and FLRT1 by biochemical approaches we next sought to study the functional consequences in a physiological setting. Given the evidence for regulated expression and function of FLRT3 in neuronal cell types [8], [9], [10] and the known role of FGFR signalling in neuronal function [15], [16], [17], [18] we elected to study the induction of neurite outgrowth in the SH-SY5Y cell line [19], [20]. Confluent cells were transfected with GFP (control) and either FLRT1 or Y3F-FLRT1 alone or in conjunction with FGFR1. Cell morphology and neuronal characteristics were then analysed in a variation of the Scholl analysis [21] (Figure S2). Cells were designated as polar, bipolar, pyramidal or multi-polar depending upon cell shape and the number of primary processes.

**Figure 3:**
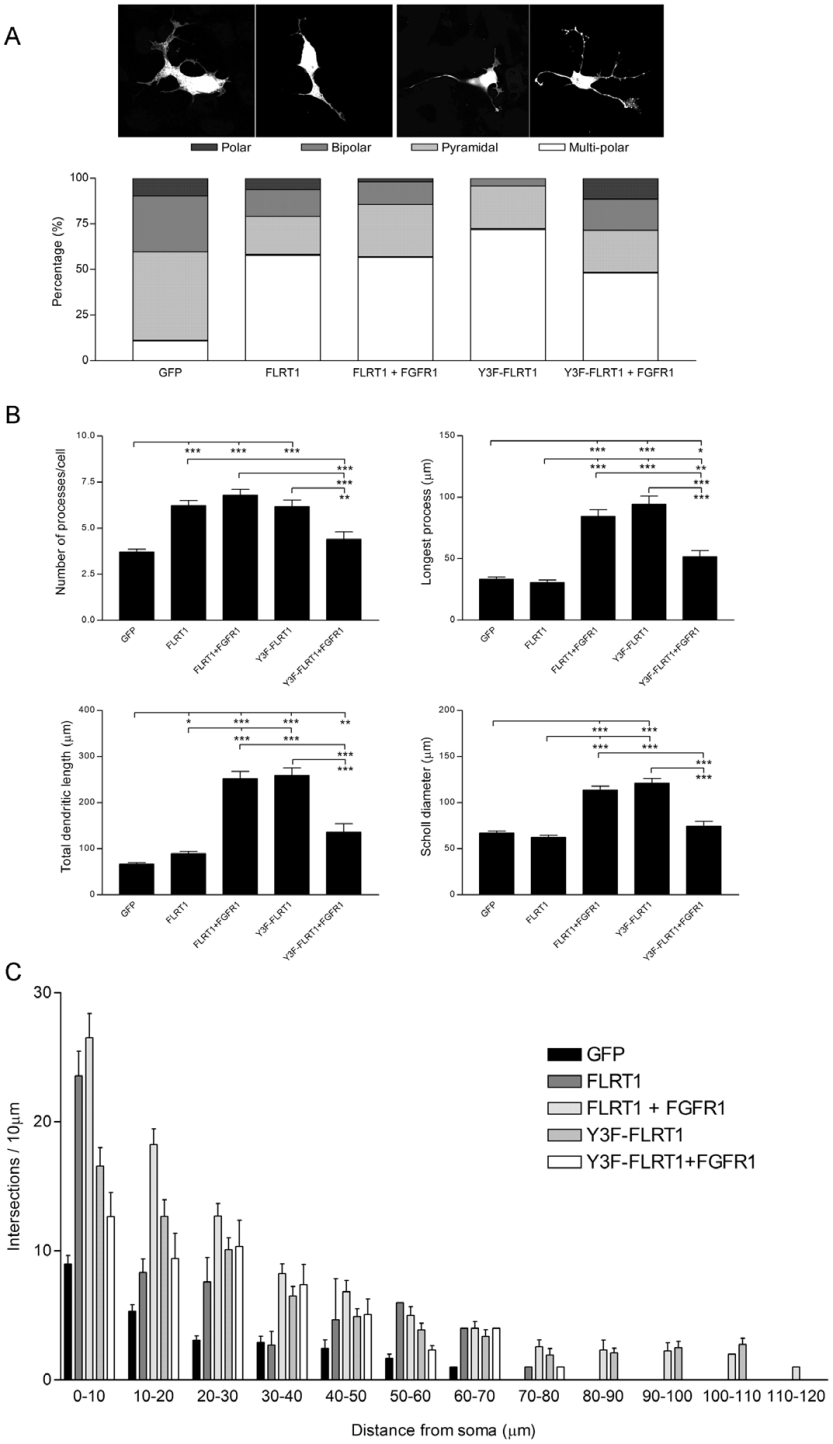
FLRT1 promotes neurite outgrowth in vitro. SH-SY5Y cells 48 hrs after transfection of either FLRT1-HA or Y3F-FLRT1-HA alone or co-transfected with FGFR1, as indicated, were stained for FLRT1 (using anti-HA) prior to morphological analysis A) Cells were assigned a ‘morphology’ based on the number of large diameter processes (>5mm in length) and cell shape as typified by the examples shown and B) several characteristic neuronal parameters were determined, such as number of processes, length of the longest process, total dendritic length and maximum cell diameter (Scholl diameter) Data in A) and B) was derived from •100 cells from at least 3 independent experiments C) the complexity of the dendritic arbour (Table S1) in a radial profile from the soma, was measured in a variation of the Scholl analysis (Sholl, 1953; see Figure S2) Data are represented as mean ± sem, n • 50 cells from at least 3 independent experiments Statistical significance in all instances used non-parametric Kruskal-Wallis and post hoc Dunns test (***p<0001, **p<001, *p<005) Images were acquired on a Leica confocal microscope and Image J was used to process stacked confocal sections to allow data determination.

Expression of either FLRT1 or Y3F-FLRT1 alone or co-expression with FGFR1 results in a significantly higher proportion of multi-polar cells and concomitant decreased numbers of other neuron types (Figure 3A). This is consistent with the significantly increased number of processes observed under these conditions (Figure 3B, upper left panel).

Expression of FLRT1 alone results in significant increases in the number of processes per cell (∼68%) and the total dendritic length (∼31%) compared to GFP control (Figure 3B). There was no significant difference in either the length of the longest process or the maximum diameter of the cell (including processes – Scholl diameter). This is consistent with the reported actions of FLRT expression on neuronal function [8], [10] possibly mediated via a cell adhesion mechanism.

In contrast, both FLRT1/FGFR1 co-expression and Y3F-FLRT1 expression results in comparable and significant increases not only in the number of processes (∼83% and ∼66%, respectively) and the total dendritic length (∼267% and ∼277%, respectively), but also in the length of the longest process (∼153% and ∼183%, respectively) and the Scholl diameter (∼70% and ∼81%, respectively) compared to GFP control (Figure 3B). Comparison of FLRT1 and Y3F-FLRT1 showed a significant increase in all parameters except the number of processes (∼68% and ∼66%, respectively). Neurite outgrowth was completely blocked in the presence of inhibitors of the MAPK pathway (U0126), FGFR1 (SU5402) and SFKs (SU6656) (Figure S6).

There was a significant increase in the length of the longest process and total dendritic length when Y3F-FLRT1 and FGFR1 were co-expressed (∼55% and ∼98%, respectively) compared to GFP. Y3F-FLRT1/FGFR1 co-expression exhibited significant reductions in all parameters when compared to either FLRT1/FGFR1 or Y3F-FLRT1 expressing cells whilst in contrast there was a significant decrease in process number and increase in longest process when Y3F-FLRT1/FGFR1 were compared to FLRT1 expressing cells (Figure 3B).

Dendritic architecture, the number and frequency of intersections and spines, is critical to the morphology and function of neuronal cells. Using a variation on the Scholl analysis, the number of spines and intersections was quantified as a function of distance from the soma (per 10mm). Despite some significant increases in dendritic complexity (Table S1), particularly for FLRT1 (0–10mm), Y3F-FLRT1 (0–30mm) and FLRT1/FGFR1 (0–40mm) expressing cells, there was no change in the radial profile of dendritic complexity with the peak remaining in the first 10mm and steadily declining with increased distance from the cell body (Figure 3C). The rather surprising results we observed when comparing FLRT1 only expression with Y3F-FLRT1/FGFR1 co-expression suggest that up-regulation of FGFR1 can counteract the effect of deregulating FLRT1 phosphorylation. Whilst suggestive of a bipartite mechanism, this remains unclear and further analysis will be required to resolve completely the functions of FLRT that are FGFR-dependent/independent.

Together these data define two features of FLRT1 action in this neuronal cell model. Dendritic architecture is regulated by FLRT1 alone whereas the length and complexity of dendrites is regulated by the signalling functions of FLRT1 acting in concert with FGFR1 activation.

### FLRT1 promotes dendritic outgrowth in primary hippocampal neurons

**Figure 4:**
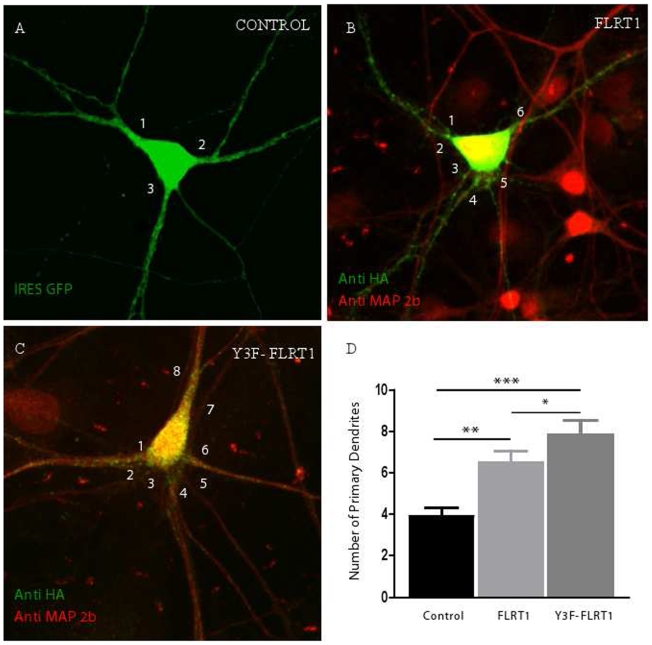
FLRT1 and Y3F-FLRT1 increase the number of primary dendrites in rat hippocampal neurons. (A–C) Representative confocal microscopic images of the number of primary dendrites in hippocampal neurons transfected with IRES GFP control vector (A), FLRT1 (B) and Y3F-FLRT1 (C) The number of primary dendrites increased when the neurons were transfected with FLRT1 and Y3F-FLRT1 compared to the control (D) Graphical representation of the average number of primary dendrites emerging from the soma under each condition There was a significant increase in primary dendrites in both the FLRT1-and Y3F-FLRT1transfected neurons compared to the control (P<00001, one-way analysis of variance and P = 00008, Kruskal-Wallis Test) Tukey's Multiple Comparison Test also revealed that there was a significant difference between control and FLRT1 (P<001, **), control and Y3F-FLRT1 (P<0001, ***) and FLRT1 and Y3F-FLRT1 (P<005, *) Data derived from several coverslips and two separate experiments.

To determine effects of FLRT1 on a primary neuron population, and confirm elevated activity of the Y3F-FLRT mutant, cultures of developing rat hippocampal neurons were transfected. These neurons have a single axon and a number of dendrites emerging from the cell body or soma (primary dendrites) and express both FGFR1 and FGFR2 (RR and IM submitted). Following transfection, dendrites were identified with anti-MAP2b and transfected cells with anti-HA antibodies and the number of dendrites projecting from cell bodies were counted. Both FLRT1 and Y3F-FLRT1 produced a statistically significant increase in numbers of primary dendrites compared to the control vector expressing GFP. Moreover, the Y3F-FLRT1 variant generated more primary dendrites than the normal FLRT1 protein (Figure 4). These data were consistent with those obtained using the SH-SY5Y neuroblastoma cell line (see above).

### Co-localization of FLRT1 and Y3F-FLRT1 with FGFR1 and Src family kinases

Having established the SH-SY5Y system as an appropriate experimental platform to study FLRT function as similar effects were observed in primary neurons, we next sought to define the spatial localisation of FLRT1, activated FGFR and activated SFKs.

**Figure 5:**
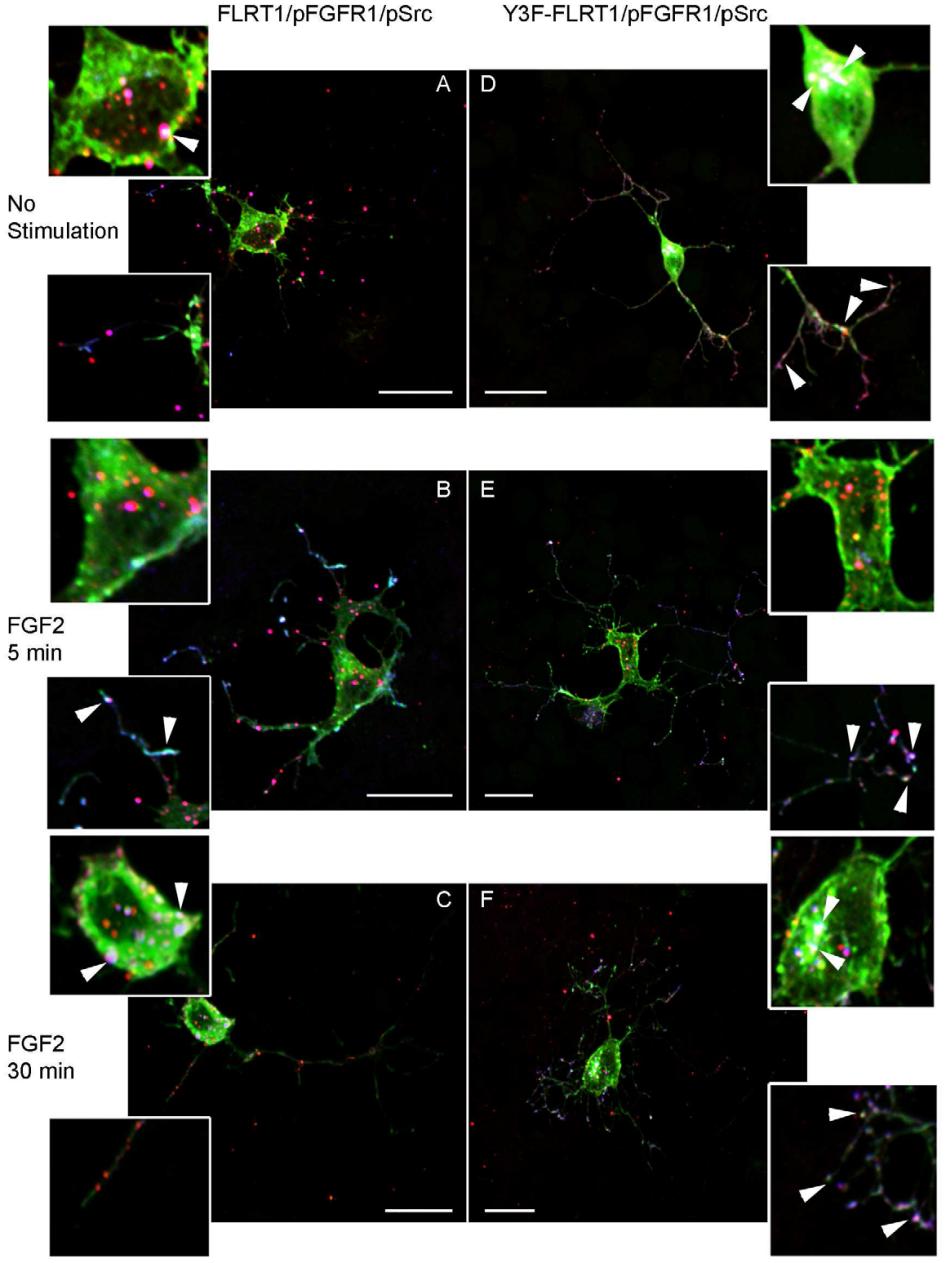
Differential trafficking and localization of activated FGFR1 by FLRT1 and Y3F-FLRT1. A–F) Representative confocal microscope images of SH-SY5Y cells grown to confluency on coverslips and co-transfected with FGFR1, and either FLRT1-HA (A–C) or Y3F-FLRT1-HA (D–F) Cells were fixed with either no stimulation, A) and D), or after exposure to FGF2 (20ng/ml) in the presence of heparin (10mg/ml) for 5 min, B) and E), or 30 min, C) and F) Cells were stained with anti-pY766FGFR1 (pFGFR1, red), anti-HA (FLRT1 and Y3F-FLRT1, green) and anti-pY416Src (pSrc, blue) Merged images (and inset magnified images of cell body and neurites) demonstrated co-localization of FLRT1/FGFR1 (yellow), FLRT1/pSrc (cyan), pFGFR1/pSrc (magenta) and FLRT1/pFGFR1/pSrc (white, white arrows) Images were acquired on a Leica confocal microscope and processed using Image J and Adobe Photoshop 60 and represent at least 3 independent experiments• Scale bar: 20mm.

FLRT1 and Y3F-FLRT1 localize in a similar manner to the plasma membrane, intracellular vesicles and punctate vesicular staining along the length of processes and the terminal end buds of processes in contact with other cells (Figure 5A and D and Figures S4 and S5) in agreement with previous reports of FLRT3 localization in neurons [8].

In cells co-expressing FLRT1 and FGFR1, activated receptor (as determined by phosphorylation of Y766; pFGFR1) localizes to vesicular structures within the cell body and the central and peripheral regions of processes; contrasting with FGFR1 only expressing cells where pFGFR1 and activated Src remain predominantly within the cell body (Figure S3). There was a small amount of pFGFR1 and FLRT1 association confined to the cell body membrane and the central regions of processes (Figure 5A and Figure S4A).

Following FGF2 (5min) stimulation, pFGFR1 localization was still observed in vesicular structures within the cell body and the central and peripheral regions of processes and prominently co-localized with both FLRT1 and pSFK (as determined by Y416 phosphorylation) in the peripheral regions of processes (Figure 5B and see Figure S4B). Prolonged (30min) stimulation with FGF2 results in transport of the majority of pFGFR to intracellular vesicular structures within the cell body where it co-localizes with FLRT1 and pSFK (Figure 5C and Figure S4C).

Striking differences were observed when Y3F-FLRT1 was co-expressed with FGFR1. pFGFR co-localizes with Y3F-FLRT1 and pSFK in both intracellular vesicles within the cell body and the central and peripheral regions of processes. Very little pFGFR co-localization with pSFK was observed independently of Y3F-FLRT1, contrasting sharply with observations from FLRT1-expressing cells. All of pFGFR, Y3F-FLRT1 and pSFK remained co-localized in the central and peripheral regions of the processes, and to a lesser degree within the cell body (Figure 5D–F and Figure S4D–F). In this respect cells co-expressing Y3F-FLRT1 (Figure 5D) closely resemble cells co-expressing FLRT1 and FGFR1 subjected to FGF stimulation (Figure 5C).

Collectively these data from a neuronal cell line confirm our biochemical observations. Stimulation of cells by FGF ligand results in activation of SFK(s) in peripheral locations and co-localisation of pSFK, pFGFR and FLRT1 which traffic to the cell body. This resembles our previous study of FGFR activation in MEFs in which FGF stimulation results in peripheral co-activation of FGFR and Src phosphorylation. In MEFs, activated Src remains confined to the periphery whilst FGFR is trafficked to perinuclear Rab5+ve vesicles [22]. In the FLRT1 expression model employed here, activated FGFR1 is also trafficked from the periphery to perinuclear locations but FLRT1 is never associated with Rab5+ve vesicles Figure S5).

In the case of Y3F-FLRT1 the 3 molecules are “pre-localized” in intracellular perinuclear vesicles consistent with the ability of Y3F-FLRT1 to activate FGFR1 signalling via a SFK-mediated mechanism.

## Discussion

In this report we have studied the functional interaction between the FGFR and its binding partner, the signal accelerator, FLRT1. We observe that tyrosine residues in the cytoplasmic domain are targets for FGFR-mediated phosphorylation and identify the residues involved. We show that mutation of the residues has the unexpected consequence of yielding a form of FLRT1 which has the property of eliciting chronic ligand-independent yet FGFR-dependent MAP Kinase activation. Y3F-FLRT1 is therefore a constitutive activator of FGFR signalling to MAP Kinase. One apparent functional consequence of FGFR-mediated phosphorylation may be to suppress the FGFR1 potentiation function of FLRT1. The FLRT/FGFR partnership therefore resembles a futile cycle in which FLRT-mediated activation of FGFR results in suppression of activation by feedback phosphorylation. Futile cycle mechanisms are classically held to exhibit super-sensitivity to input [23], [24] which is what we observe in the case of FGF. This mechanism therefore explains the ability of wild type FLRT1 to markedly accelerate the early phase of ligand-mediated signalling: this phase may represent the time required for FLRT1 to switch phosphorylation states.

The second finding in this report is that the connection between FLRT1 and FGFR1 may be moderated by a non-receptor tyrosine kinase of the Src family. FLRT1 is not a direct substrate for Src kinase but SFK activity is required for FGFR1-mediated FLRT1 phosphorylation. Conversely a SFK is implicated in the ability of Y3F-FLRT1 to activate the FGFR. We have previously shown that activated Src utilises FGFR as a substrate and pharmacological inhibition of Src resulted in prolonged ERK activation due to inhibition of signal decay [12]. This suggests the ability of Y3FFLRT1 to activate FGFR signalling is indirect arising from activation of Src kinase implying that a second consequence of FLRT1 phosphorylation is suppression of its ability to activate Src. Within this context, Src activation may be negatively regulated by one or more of the three tyrosines in the cytoplasmic domain of FLRT. We note that one target, Y600, is located adjacent to a canonical PXXP SH3 domain binding motif and it has been reported in other systems that tyrosine phosphorylation events in the proximity of an SH3 recognition motif suppress the interaction [25]. Although this is an attractive mechanism none of the single or double mutant forms of FLRT1 exhibit MAPK activation suggesting that the action of de-phosphorylated FLRT1 results from the concerted action of all three phosphorylated residues. Whilst this data is consistent with, and provides some evidence for, the involvement of a SFK in the regulation of FGFR1 via FLRT1, further studies are needed to investigate and clarify the role of SFKs in the regulation and localisation of the FLRT1-FGFR1 interdependent signalling mechanism.

We showed that the interaction between FGFR1 and FLRT1 has functional consequences in both the SH-SY5Y neuronal cell model and primary hippocampal neurons. Expression of FLRT1 alone induces a multi-polar phenotype whereas expression of Y3F-FLRT1 or co-expression of FLRT1 and FGFR1 induced both a multi-polar phenotype and elevated neurite outgrowth involving activation of the MAP Kinase pathway. The FLRT1-mediated increase in primary processes seen in the SH-SY5Y line was also seen in elevated numbers of primary dendrites in hippocampal neuron cultures and the increased signalling activity of the Y3-FLRT1 protein was reflected in a significant further increase in dendrite production above that seen with FLRT1. Importantly, perturbation of FLRT1 phosphorylation resulted in deregulated localisation of the proposed FLRT1-FGFR1-SFK signalling axis in the distal region of the neuritic arbour of SH-SY5Y cells. These findings suggest a dual role for FLRT family proteins in neuronal function: one function is mediated by the action of FLRT alone, presumably reflecting consequences of its cell adhesion properties and the second arising from activation of signal transduction pathways involving SFK(s) and FGFR1. Although we appreciate the caveat of overexpression systems and promiscuous signalling with regard to interpretation of this study in terms of physiologically relevant FGFR1-FLRT1 interactions, it should be noted that FLRT3 was up-regulated in response to both axotomisation and neuronal injury [8], [9] and it has been suggested that FLRT proteins play a role in neuronal regeneration mechanisms when they are induced and FLRT population at the membrane is elevated. Our study in primary hippocampal neurons confirm our findings in SHSY5Y cells and lend support to the hypothesized model of neuronal regeneration.

Analogous bipartite mechanisms of cell adhesion proteins on sculpting neurite morphology have previously been reported for Ig-domain cell adhesion molecules NCAM, N-cadherin [26], neurofascin [27] as well as other leucine-rich repeat adhesion molecules [28]. This dual role of FLRTs may explain the lack of alteration of FGF target gene expression in early mouse embryos homozygous for a mutation in the FLRT3 gene [29] with the phenotypes seen in these embryos due to FLRT function independent of FGFR signalling. The dual action of adhesion molecules and FGFR signalling may represent key mechanisms for refining the spatial and temporal dynamics of FGFR signalling during neuronal development and function.

## Materials and Methods

### Cell culture and transfection

HEK 293T cells were cultured at 37°C, 5% CO 2 in Dulbecco's modified Eagle medium (DMEM, Invitrogen) supplemented with 2mM L-glutamine (Invitrogen), 0.1 mg/ml streptomycin, 0.2 U/ml penicillin (Sigma), 1mM sodium pyruvate (Sigma), 10% (v/v) fetal calf serum (FCS -Labtech International). SH-SY5Y cells were cultured in RPMI 1640 (Invitrogen) supplemented with 2mM L-glutamine (Invitrogen), nonessential amino acids (GIBCO), 0.1 mg/ml streptomycin, 0.2 U/ml penicillin (Sigma) and 10% (v/v) FCS. For FGF2 stimulation, cells were serum starved by replacing media with Krebs HEPES Buffer (KHB), and incubated at 37°C (1 hour) prior to the addition of either 20ng/ml recombinant human FGF2 (in the presence of 10mg/ml heparin, stimulated) or vehicle (KHB, non-stimulated) for the indicated times.

Using the C-terminal HA-tagged FLRT1 cDNA [4] as a template, cDNAs of FLRT1 with Y600 (Y600F-FLRT1) or Y633 (Y633F-FLRT1) and both tyrosines (Y2F-FLRT1) mutated to phenylalanine were constructed by PCR with the open reading frame reconstructed using a unique Xba1 site present between these residues. FLRT1 cDNAs with either the Y671 (Y671F-FLRT1) or three tyrosines, Y600, Y633 and Y671 (Y3F-FLRT1), mutated were constructed by ‘Quickchange’ mutagenesis (Stratagene) on wild-type C-terminal HA tagged FLRT1 and Y2F-FLRT1, respectively. Expression constructs for mammalian cells were in pCDNA3.1 (Invitrogen). Plasmids encoding human FGFR1 [30], HA-tagged FLRT1 constructs or Src constructs (M.Frame, Beatson Institute, Glasgow), were transiently transfected into HEK293T cells by the DNA/CaPO4 precipitation method, incubated on the cells overnight and cells washed the following morning with Ultracho (Cambrex) and the media replaced with 2ml of Ultracho. Recombinant proteins were expressed for 48 hrs.

### Cell lysis, immunoprecipitation (IP) and western blot analysis (IB)

HEK 293T cells were lysed with RIPA buffer (supplemented with 1mM Na3VO4, 50mM NaF, 25mM b-glycerophosphate and 1 tablet of complete protease inhibitor cocktail (Roche) per 10ml of buffer, pH 8.0). Aliquots of whole cell lysate were subjected to SDS-PAGE and the remainder subjected to immunoprecipitation by the addition of 2ml of monoclonal anti-HA (6E2-Cell Signalling) and incubation at 4oC for 1hr. Immuno-captured complexes were isolated by the addition of 20ml of protein-Asepharose fast flow (Amersham Biosciences, Inc., UK) and incubated for 30min at 4oC. Samples were washed (3×) with Tris Buffered Saline-Tween (TBS-T: 10mM Tris/HCl, pH 7.4, 75mM NaCl, 0.05% Tween-20 (v/v)) and then washed (2×) with TE buffer before bound proteins were eluted by boiling with SDS sample buffer containing 200mM DTT, pH 6.8.

Protein samples were run on 4–20% gradient SDS PAGE (Lonza) at 125V and calibrated with SeeBlue Plus2 pre-stained markers (10•l, Invitrogen). Gels were transferred to nitrocellulose membrane (Protran BA85, Schleicher and Schuell) at 200mA/gel for 1 hour on a Biometra Semi-dry transfer system. Membranes were blocked in TBS-T containing 5% bovine serum albumin (BSA, w/v). Primary antibodies (in TBS-T/5% BSA) were incubated with the membrane at either 4oC overnight or 1 hr at room temperature. Membranes were washed (3×15 min) in TBS-T and subsequently probed with conjugated secondary antibody (in TBS-T/5% BSA) for 45 min at room temperature. The membrane was washed (5×10 min) with TBS-T, before membranes were exposed to EZ-ECL (Geneflow) for visualization of immunoreactive proteins. Antibodies included anti-FGFR1 (C15, Santa Cruz), anti-HA (6E2, Cell Signalling), anti-phosphotyrosine cocktail of 4G10 (Upstate) and pY20 (MP Biomedicals Ltd), anti-ERK (K23-Santa Cruz) and anti-phosphoERK (E4-Santa Cruz).

### Immunofluorescence

For immunofluorescence studies, Cos-7 or SH-SY5Y cells were grown on coverslips and transfected (Fugene 6 (Roche) or Genejuice (Invitrogen), respectively) as per the manufacturer's instructions. 48 hours post transfection Cos-7 cells were fixed and permeabilised in methanol for 2 min at -20oC and re-hydrated in PBS for 15 min whilst SH-SY5Y cells were fixed with 4% PFA and permeabilised with methanol (5 min at −20°C). Following 1hr incubation in PBS/4% BSA to reduce non-specific binding, coverslips were incubated with primary antibody(s): anti-HA (HA.11, 1∶500, Covance); anti-HA (6E2, 1∶200); anti-FGFR1 (C15, 1∶50, Santa Cruz); anti-pY766 FGFR1 (Tyr766m, 1∶100, Santa Cruz); anti-pY416 Src (2101S, 1∶100, Cell Signalling); in PBS-T/4% BSA for 1hr. Coverslips were washed 3 times in PBS and incubated with secondary antibody(s): anti-mouse Alexa 555 antibody (1∶1000); anti-rabbit Alexa 488 antibody (1∶1000); anti-goat Alexa 594 (1∶200); anti-rabbit Alexa 647 (1∶250); anti-mouse FITC (1∶400); anti-rabbit FITC (1∶200); anti-mouse Texas Red (1∶200) (all from Molecular Probes), in PBS-T/4% BSA for 1hr and washed 3 times in PBS containing 0.1% Tween-20, once in dH2O. Coverslips were mounted using Vectastain (Cos-7)/Mowiol (SH-SY5Y) mounting media and images obtained as sections by confocal microscope (Leica).

### Transfection of cultured hippocampal neurons

Primary hippocampal cultures were prepared from embryonic day 18 Sprague-Dawley rats. Hippocampi were dissociated with trypsin (5mg/ml for 15 min at 37oC; Worthington), triturated and plated onto coverslips coated with poly-D-lysine (50 mg/ml) and laminin (20 mg/ml) at a density of 90,000 neurons per coverslip. Neurons were incubated at 37oC in 5% CO2 in Neurobasal medium supplemented with B27, glutamax and penicillin/streptomycin (all Gibco). After 5 days in culture, neurons were transfected with 1 •g/ml plasmid DNA using Lipofectamine 2000 (Invitrogen). They were cultured for a further 9 days, fixed in 4% (w/v) paraformaldehyde phosphate-buffered saline (PBS) and permeablized with 0.25% (v/v) Triton X-100 in PBS.

After blocking with 10% (w/v) bovine serum albumin (BSA) in PBS, coverslips were incubated for 2 hours at room temperature with one or more of the following antibodies in 3% BSA: mouse anti-HA (1∶400; Abcam) and rabbit anti-MAP2b (1∶500; Abcam). After washing, cells were incubated for 1 hour at room temperature in 3% BSA containing the appropriate secondary antibodies (conjugated to Alexa 568 or Alexa 633; Invitrogen). Coverslips were mounted for viewing on an Olympus FV1000 confocal microscope. Statistical analyses were performed with a Kruskal-Wallis Test for one-way variance and with Tukey's Multiple Comparison Test.

## Supporting Information

Figure S1 FGF2-dependent phosphorylation of FLRT1. HEK 293T cells were transfected with the indicated constructs and pre-incubated with pharmacological inhibitors where indicated (1hr). Following 20 min stimulation with FGF2 (20ng/ml) and heparin (10mg/ml) cell lysates were A) immunoprecipitated with anti-HA (IP: HA) and subsequently blotted with anti-phosphotyrosine (Blot: pY) or anti-HA (Blot: HA). Whole cell lysate fractions (WCL) were probed with anti-FGFR1 (Blot: FGFR1) to control for protein expression B) Immunoprecipitated with anti-HA (IP: HA) and subsequently blotted with anti-phosphotyrosine (Blot: pY), anti-HA (Blot: HA) or anti-FGFR1 (Blot: Flg). Whole cell lysate fractions (WCL) were probed with anti-phosphoERK (Blot: pERK) or anti-ERK (Blot: ERK). Data in A) and B) are representative of 3 independent experiments. Densitometric analysis (mean +/− sem, n = 3) and adjusted for FLRT1 expression and normalised to FLRT1 phosphorylation when wild type FLRT1 is co-expressed with FGFR1 (**p<0.01, *p<0.05 non-parametric one way ANOVA). C) 293T cells were co-transfected with either a constitutively active (KA) or kinase dead (KD) c-Src. Cells were stimulated with FGF2 and heparin (as above). Cell lysates were immunoprecipitated with anti-HA (IP: HA) and subsequently probed with anti-phosphotyrosine (Blot: pY) or anti-HA (Blot: HA). Whole cell lysates (WCL) were probed for anti-phosphoERK (Blot: pERK) and anti-ERK (Blot: ERK). Data are representative of two independent experiments.
(0.97 MB TIF)

Figure S2 Variation on the Scholl analysis. Neuronal morphology was assessed using Image J to determine the following parameters. The number of processes >5mm in length (*) were counted; the length of processes was determined by freehand tracing (green line); the total dendritic length is the sum of all measured processes; the maximum diameter (Scholl diameter - solid red line) was measured; and dendritic complexity assessed by counting the number of spines and intersections along a process, every 10µm from the cell soma (red dotted circles). Scale bar = 10µm.
(5.70 MB TIF)

Figure S3 Transfected FGFR1 associates with pSrc but remains predominantly within the cell body. Confocal analysis of SH-SY5Y cells grown to confluence and transfected with FGFR1. Following 48 hrs of expression, cells were fixed following either no stimulation or after exposure to FGF2 (20ng/ml) and heparin (10mg/ml) for 5 and 30 min. Cells were stained with anti-pY766FGFR1 (pFGFR, red) and anti-pY416Src (pSrc, blue) and merged to show co-localization of pFGFR/pSrc (magenta). Images were acquired on a Leica confocal microscope and processed using Image J and Adobe Photoshop 6.0 and represent 2 independent experiments. Scale bar: 20µm.
(2.25 MB TIF)

Figure S4 Localization of FLRT1, pFGFR1 and pSrc. Confocal images of SH-SY5Y cells transfected with either FLRT-HA (A–C) or Y3F-FLRT1-HA (D–F). Following 48 hrs of expression, cells were fixed following either no treatment (A and D) or following FGF2 stimulation (B, C, E, F). Cells were stained for anti-HA (Y3F-FLRT1, green), anti-pY766FGFR1 (pFGFR, red) or anti-pY416Src (pSrc, blue). Merged images demonstrate co-localization of Y3F-FLRT1/pFGFR (yellow), Y3F-FLRT1/pSrc (cyan) and pFGFR/pSrc (magenta). Images were acquired on a Leica confocal microscope and processed with Image J and Adobe Photoshop 6.0 and represent three independent experiments. Scale bar: 10µm.
(3.08 MB TIF)

Figure S5 Neither FLRT1 nor Y3F-FLRT1 co-localize with Rab5. Confocal sections of SH-SY5Y cells grown on coverslips and transfected with A) FLRT1-HA or B) Y3F-FLRT1-HA. Cells were fixed following either no stimulation or after exposure to FGF2 (20ng/ml) and heparin (10mg/ml) for either 5 or 30 min. Cells were stained with anti-HA (FLRT1 and Y3F-FLRT1, red) and anti-Rab5 (S19, 1∶300, Santa Cruz, green). Merged images demonstrated no co-localization of FLRT1/Rab5 or Y3F-FLRT1/Rab5. Images were acquired on a Leica confocal microscope and processed using Image J and Adobe Photoshop 6.0 and represent 3 independent experiments. Scale bar: 20µm.
(5.74 MB TIF)

Figure S6 Pharmacological Inhibition of neurite outgrowth in the SH-SY5Y cells. SH-SY5Y cells were transfected with GFP (control), FLRT1-HA or Y3F-FLRT1-HA and simultaneously treated with inhibitors (U0126, MEK inhibitor; SU5402, FGFR inhibitor; SU6656, SFK inhibitor; SB203580, p38 MAPK inhibitor). After 48 hours of treatment, cells were analysed by direct confocal microscopy (in the case of GFP-expressing cells) or stained for anti-HA (FLRT1 and Y3F-FLRT1, green, middle and right hand panels respectively). Inhibition of the p38 MAPK had no visible effect on either cell morphology or the ability of either FLRT1 or Y3F-FLRT1 to promote neurite outgrowth. Inhibition of either MEK/ERK (U0126) or SFK (SU6656) signalling resulted in a complete loss of neurite outgrowth from the cells consistent with evidence in the literature that has implicated SFKs and ERK as critical processes for neurite extension. In line with our model of FLRT1-mediated modulation of FGFR1 signalling, inhibition of the receptor by SU5402 prevented neurite outgrowth in both the control and FLRT1 expressing cells. In contrast, neurite outgrowth in cells expressing Y3F-FLRT1, which is receptor-independent for activation of ERK, was unaffected by SU5402 treatment. Images were acquired on a Leica confocal microscope, Image J and Adobe Photoshop 6.0 were used to process captured data.
(2.95 MB TIF)

Table S1 Statistical summary of SH-SY5Y dendritic architecture analysis by non-parametric Kruskal-Wallis and post hoc Dunns test.
(0.05 MB DOC)
