# Supplementary figures and images for: Critical Role of FLRT1 Phosphorylation in the Interdependent Regulation of FLRT1 Function and FGF Receptor Signalling
Source: PLoS One. 2010 Apr 22;5(4):e10264. doi: 10.1371/journal.pone.0010264 (PMC2858647; doi:10.1371/journal.pone.0010264)

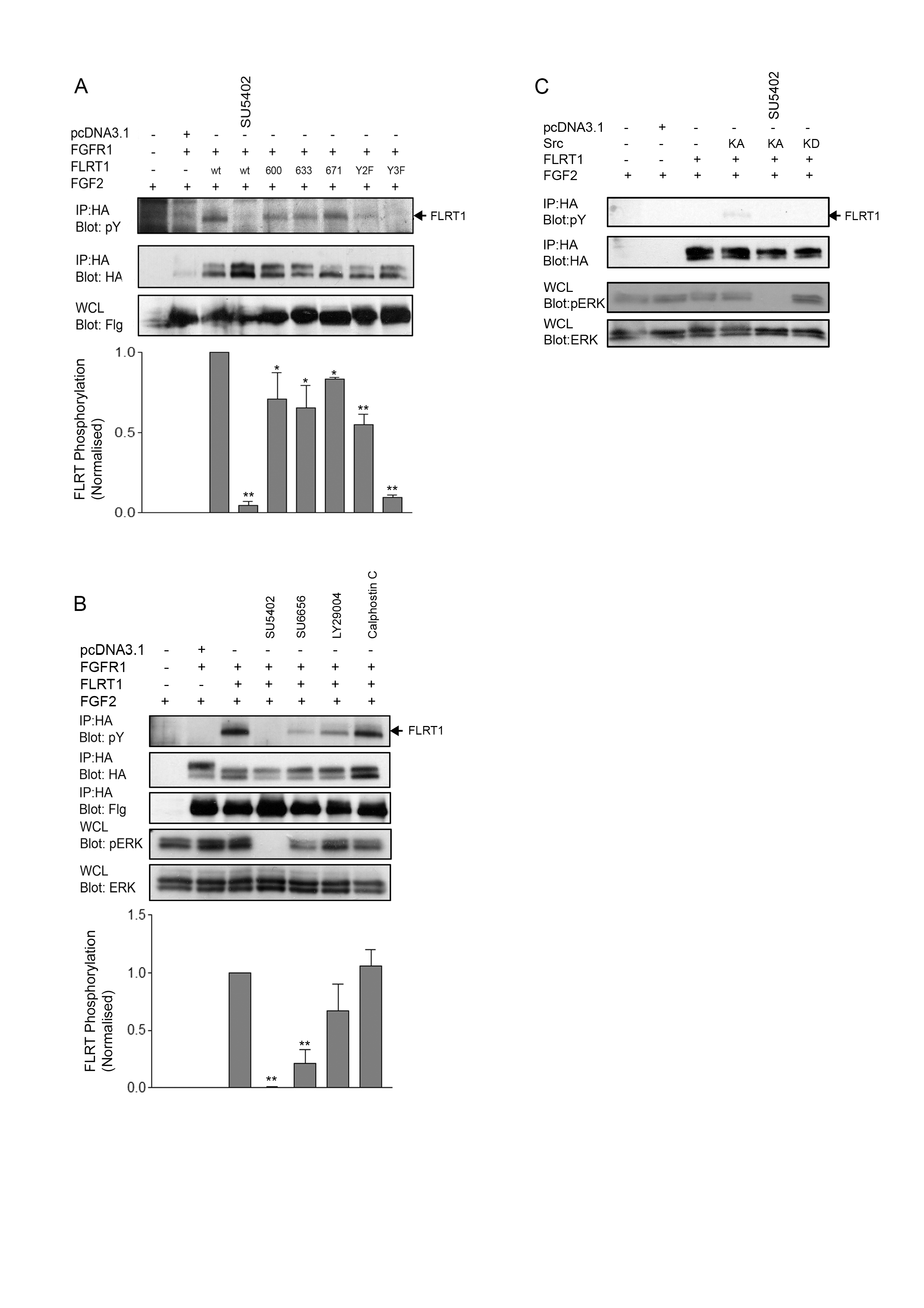

Supplement: Figure S1 — FGF2-dependent phosphorylation of FLRT1. HEK 293T cells were transfected with the indicated constructs and pre-incubated with pharmacological inhibitors where indicated (1hr). Following 20 min stimulation with FGF2 (20ng/ml) and heparin (10mg/ml) cell lysates were A) immunoprecipitated with anti-HA (IP: HA) and subsequently blotted with anti-phosphotyrosine (Blot: pY) or anti-HA (Blot: HA). Whole cell lysate fractions (WCL) were probed with anti-FGFR1 (Blot: FGFR1) to control for protein expression B) Immunoprecipitated with anti-HA (IP: HA) and subsequently blotted with anti-phosphotyrosine (Blot: pY), anti-HA (Blot: HA) or anti-FGFR1 (Blot: Flg). Whole cell lysate fractions (WCL) were probed with anti-phosphoERK (Blot: pERK) or anti-ERK (Blot: ERK). Data in A) and B) are representative of 3 independent experiments. Densitometric analysis (mean +/− sem, n = 3) and adjusted for FLRT1 expression and normalised to FLRT1 phosphorylation when wild type FLRT1 is co-expressed with FGFR1 (**p<0.01, *p<0.05 non-parametric one way ANOVA). C) 293T cells were co-transfected with either a constitutively active (KA) or kinase dead (KD) c-Src. Cells were stimulated with FGF2 and heparin (as above). Cell lysates were immunoprecipitated with anti-HA (IP: HA) and subsequently probed with anti-phosphotyrosine (Blot: pY) or anti-HA (Blot: HA). Whole cell lysates (WCL) were probed for anti-phosphoERK (Blot: pERK) and anti-ERK (Blot: ERK). Data are representative of two independent experiments. (0.97 MB TIF) [file pone.0010264.s001.tif]

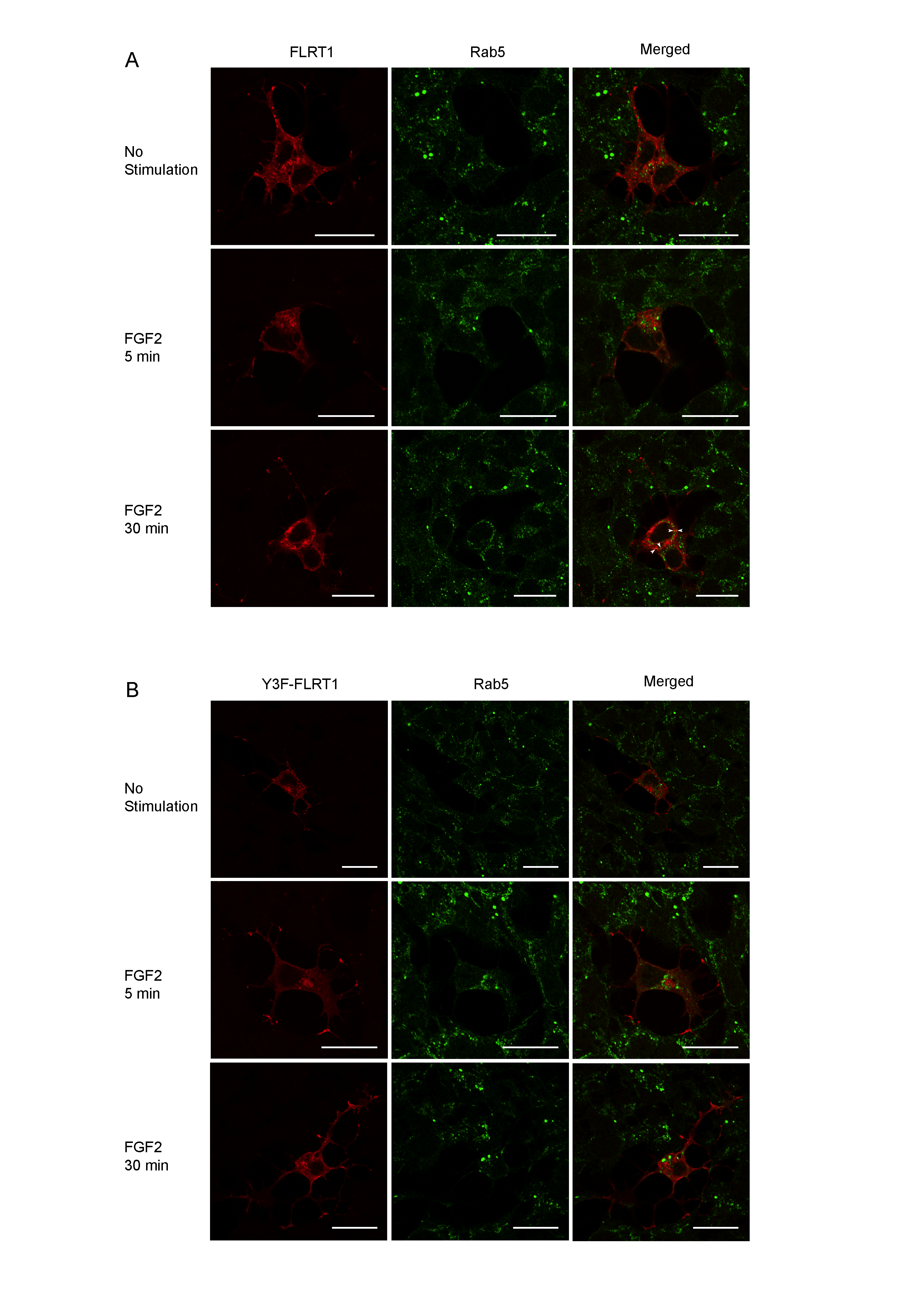

Supplement: Figure S2 — Variation on the Scholl analysis. Neuronal morphology was assessed using Image J to determine the following parameters. The number of processes >5mm in length (*) were counted; the length of processes was determined by freehand tracing (green line); the total dendritic length is the sum of all measured processes; the maximum diameter (Scholl diameter - solid red line) was measured; and dendritic complexity assessed by counting the number of spines and intersections along a process, every 10µm from the cell soma (red dotted circles). Scale bar = 10µm. (5.70 MB TIF) [file pone.0010264.s002.tif]

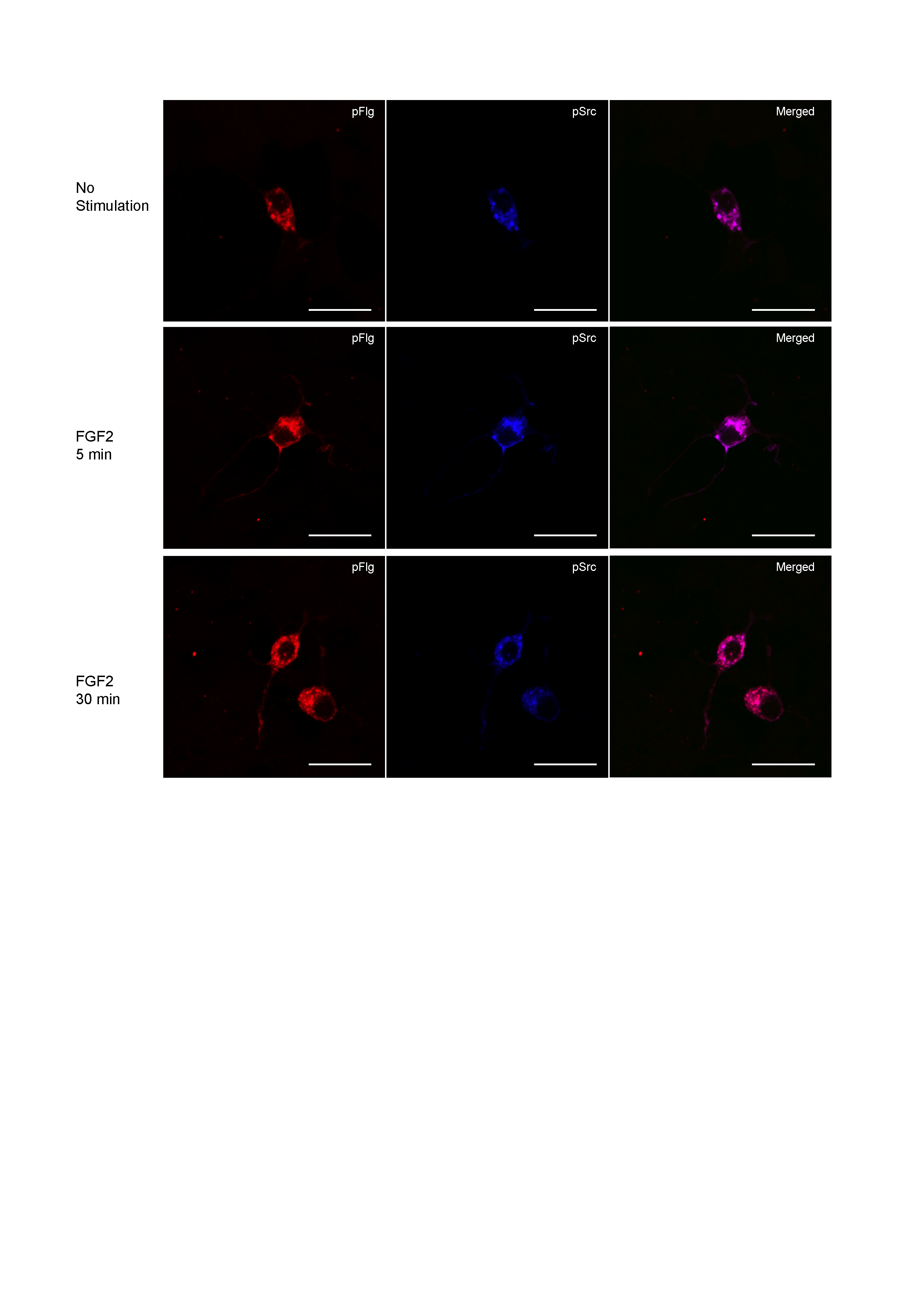

Supplement: Figure S3 — Transfected FGFR1 associates with pSrc but remains predominantly within the cell body. Confocal analysis of SH-SY5Y cells grown to confluence and transfected with FGFR1. Following 48 hrs of expression, cells were fixed following either no stimulation or after exposure to FGF2 (20ng/ml) and heparin (10mg/ml) for 5 and 30 min. Cells were stained with anti-pY766FGFR1 (pFGFR, red) and anti-pY416Src (pSrc, blue) and merged to show co-localization of pFGFR/pSrc (magenta). Images were acquired on a Leica confocal microscope and processed using Image J and Adobe Photoshop 6.0 and represent 2 independent experiments. Scale bar: 20µm. (2.25 MB TIF) [file pone.0010264.s003.tif]

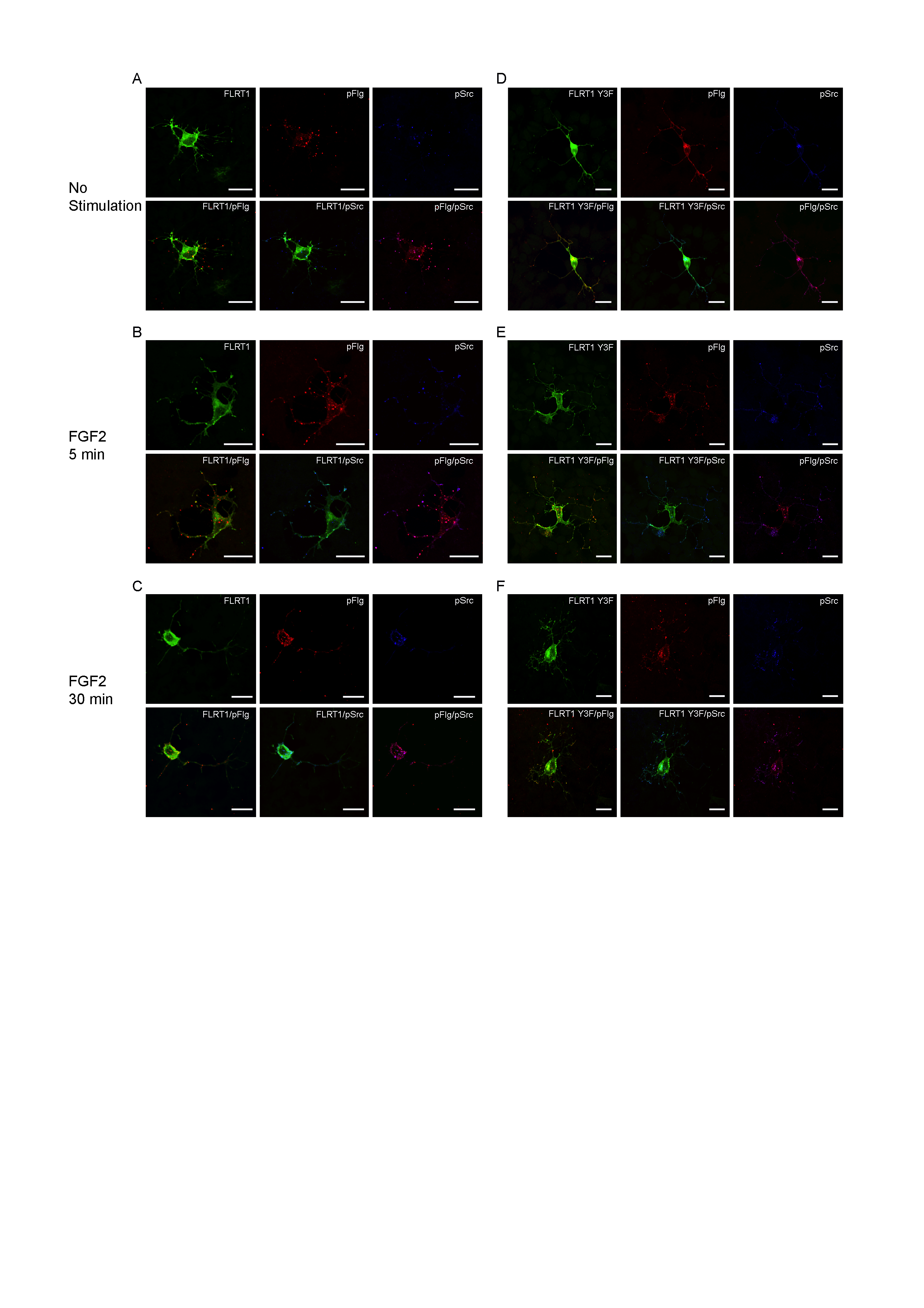

Supplement: Figure S4 — Localization of FLRT1, pFGFR1 and pSrc. Confocal images of SH-SY5Y cells transfected with either FLRT-HA (A–C) or Y3F-FLRT1-HA (D–F). Following 48 hrs of expression, cells were fixed following either no treatment (A and D) or following FGF2 stimulation (B, C, E, F). Cells were stained for anti-HA (Y3F-FLRT1, green), anti-pY766FGFR1 (pFGFR, red) or anti-pY416Src (pSrc, blue). Merged images demonstrate co-localization of Y3F-FLRT1/pFGFR (yellow), Y3F-FLRT1/pSrc (cyan) and pFGFR/pSrc (magenta). Images were acquired on a Leica confocal microscope and processed with Image J and Adobe Photoshop 6.0 and represent three independent experiments. Scale bar: 10µm. (3.08 MB TIF) [file pone.0010264.s004.tif]

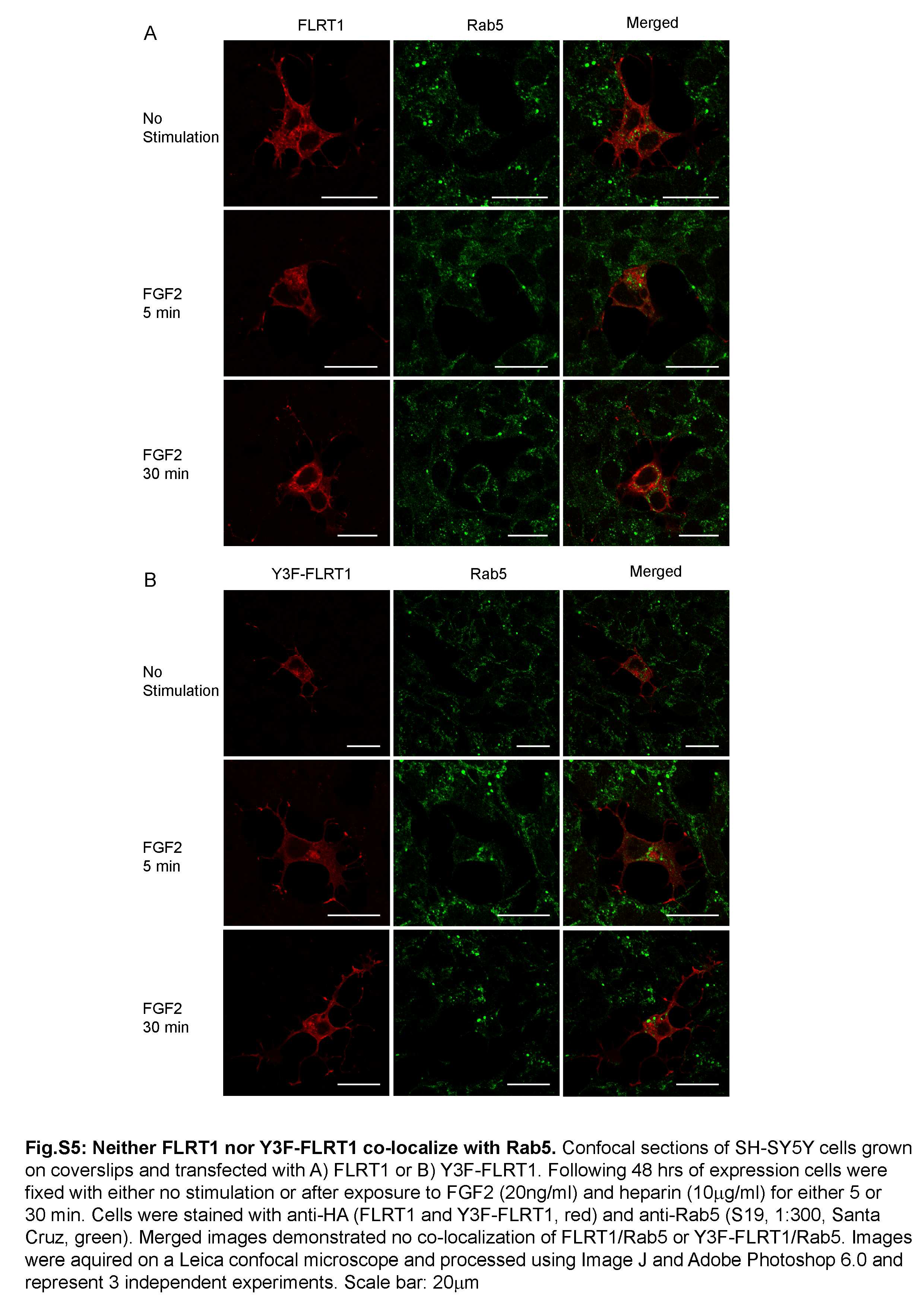

Supplement: Figure S5 — Neither FLRT1 nor Y3F-FLRT1 co-localize with Rab5. Confocal sections of SH-SY5Y cells grown on coverslips and transfected with A) FLRT1-HA or B) Y3F-FLRT1-HA. Cells were fixed following either no stimulation or after exposure to FGF2 (20ng/ml) and heparin (10mg/ml) for either 5 or 30 min. Cells were stained with anti-HA (FLRT1 and Y3F-FLRT1, red) and anti-Rab5 (S19, 1∶300, Santa Cruz, green). Merged images demonstrated no co-localization of FLRT1/Rab5 or Y3F-FLRT1/Rab5. Images were acquired on a Leica confocal microscope and processed using Image J and Adobe Photoshop 6.0 and represent 3 independent experiments. Scale bar: 20µm. (5.74 MB TIF) [file pone.0010264.s005.tif]

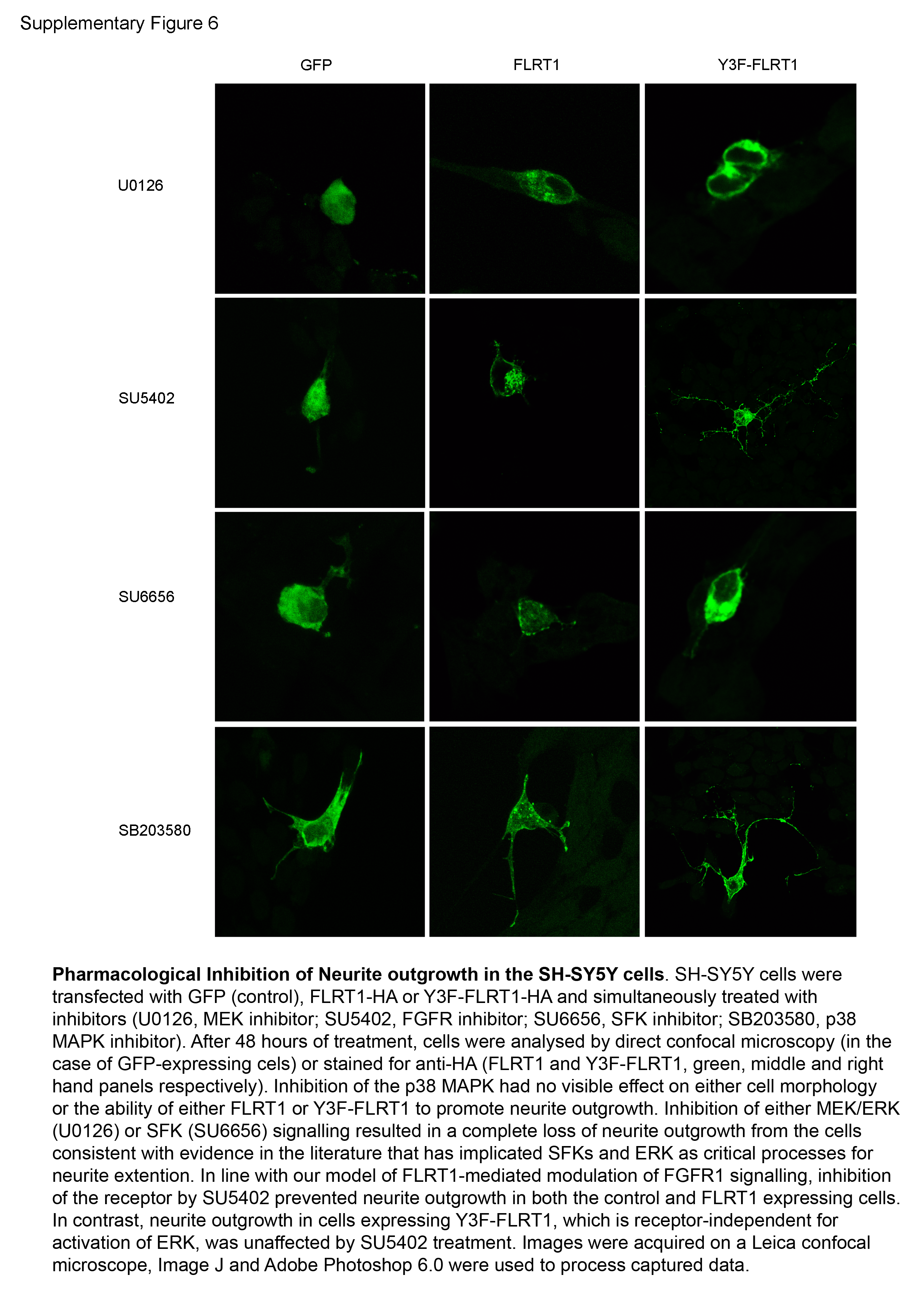

Supplement: Figure S6 — Pharmacological Inhibition of neurite outgrowth in the SH-SY5Y cells. SH-SY5Y cells were transfected with GFP (control), FLRT1-HA or Y3F-FLRT1-HA and simultaneously treated with inhibitors (U0126, MEK inhibitor; SU5402, FGFR inhibitor; SU6656, SFK inhibitor; SB203580, p38 MAPK inhibitor). After 48 hours of treatment, cells were analysed by direct confocal microscopy (in the case of GFP-expressing cells) or stained for anti-HA (FLRT1 and Y3F-FLRT1, green, middle and right hand panels respectively). Inhibition of the p38 MAPK had no visible effect on either cell morphology or the ability of either FLRT1 or Y3F-FLRT1 to promote neurite outgrowth. Inhibition of either MEK/ERK (U0126) or SFK (SU6656) signalling resulted in a complete loss of neurite outgrowth from the cells consistent with evidence in the literature that has implicated SFKs and ERK as critical processes for neurite extension. In line with our model of FLRT1-mediated modulation of FGFR1 signalling, inhibition of the receptor by SU5402 prevented neurite outgrowth in both the control and FLRT1 expressing cells. In contrast, neurite outgrowth in cells expressing Y3F-FLRT1, which is receptor-independent for activation of ERK, was unaffected by SU5402 treatment. Images were acquired on a Leica confocal microscope, Image J and Adobe Photoshop 6.0 were used to process captured data. (2.95 MB TIF) [file pone.0010264.s006.tif]
