# Supplementary material for: Critical Role of FLRT1 Phosphorylation in the Interdependent Regulation of FLRT1 Function and FGF Receptor Signalling
Source: PLoS One. 2010 Apr 22;5(4):e10264. doi: 10.1371/journal.pone.0010264 (PMC2858647; doi:10.1371/journal.pone.0010264)
Supplement: Table S1 — Statistical summary of SH-SY5Y dendritic architecture analysis by non-parametric Kruskal-Wallis and post hoc Dunns test. (0.05 MB DOC) [file pone.0010264.s007.doc]

**Supplementary Table1**: Statistical summary of SH-SY5Y dendritic architecture

|  | Distance from Soma (μm) | | | | | | | | | | |
| --- | --- | --- | --- | --- | --- | --- | --- | --- | --- | --- | --- |
| Dunn's Multiple Comparison Test | 0-10 |  |  | 10-20 |  |  | 20-30 |  |  | 30-40 |  |
|  |  |  |  |  |  |  |  |  |  |  |  |
| GFP vs  FLRT1 | P < 0.001 | *** |  | P > 0.05 | ns |  | P > 0.05 | ns |  | P > 0.05 | ns |
| GFP vs FLRT1+FGFR1 | P < 0.001 | *** |  | P < 0.001 | *** |  | P < 0.001 | *** |  | P < 0.001 | *** |
| GFP vs  Y3F-FLRT1 | P < 0.001 | *** |  | P < 0.001 | *** |  | P < 0.001 | *** |  | P > 0.05 | ns |
| GFP vs  Y3F-FLRT1+FGFR1 | P > 0.05 | ns |  | P > 0.05 | ns |  | P < 0.05 | * |  | P > 0.05 | ns |
|  |  |  |  |  |  |  |  |  |  |  |  |
| FLRT1 vs  FLRT1+FGFR1 | P > 0.05 | ns |  | P < 0.001 | *** |  | P < 0.01 | ** |  | P < 0.05 | * |
| FLRT1 vs  Y3F-FLRT1 | P > 0.05 | ns |  | P > 0.05 | ns |  | P > 0.05 | ns |  | P > 0.05 | ns |
| FLRT1 vs  Y3F-FLRT1+FGFR1 | P < 0.001 | *** |  | P > 0.05 | ns |  | P > 0.05 | ns |  | P > 0.05 | ns |
|  |  |  |  |  |  |  |  |  |  |  |  |
| FLRT1+FGFR1 vs  Y3F-FLRT1 | P < 0.01 | ** |  | P < 0.05 | * |  | P > 0.05 | ns |  | P > 0.05 | ns |
| FLRT1+FGFR1 vs  Y3F-FLRT1+FGFR1 | P < 0.001 | *** |  | P < 0.001 | *** |  | P > 0.05 | ns |  | P > 0.05 | ns |
|  |  |  |  |  |  |  |  |  |  |  |  |
| Y3F-FLRT1 vs Y3F-FLRT1+FGFR1 | P > 0.05 | ns |  | P < 0.05 | * |  | P > 0.05 | ns |  | P > 0.05 | ns |
